# Swiss Consensus on Prenatal and Early Postnatal Urinary Tract Dilation: Practical Approach and When to Refer

**DOI:** 10.3390/children11121561

**Published:** 2024-12-23

**Authors:** Atessa Bahadori, Alexandra Wilhelm-Bals, Julien Caccia, Hassib Chehade, Alexandra Goischke, Céline Habre, Daniela Marx-Berger, Samuel Nef, Oliver Sanchez, Giuseppina Spartà, Isabelle Vidal, Rodo O. von Vigier, Jacques Birraux, Paloma Parvex

**Affiliations:** 1 Nephrology Unit, Paediatric Specialties Division, Geneva University Hospitals (HUG), 1205 Geneva, Switzerland; alexandra.wilhelm-bals@hug.ch (A.W.-B.); paloma.parvex@hug.ch (P.P.); 2 Division of Paediatric Nephrology, Department of Paediatrics, The Hospital for Sick Children, University of Toronto, Toronto, ON M5G 1E8, Canada; 3 Division of Paediatric Nephrology, University Children’s Hospital, 3010 Bern, Switzerland; julien.caccia@insel.ch; 4 Paediatric Nephrology Unit, Paediatric Division, Woman-Mother-Child Department, Lausanne University Hospital (CHUV), 1011 Lausanne, Switzerland; hassib.chehade@chuv.ch; 5 Nephrology Department, University Children’s Hospital (UKBB), 4031 Basel, Switzerland; alexandra.goischke@ukbb.ch; 6 Division of Radiology, Geneva University Hospitals (HUG), 1205 Geneva, Switzerland; celine.habre@hug.ch; 7 Paediatric Nephrology, Children’s Hospital of Eastern Switzerland (OKS), 9006 St. Gallen, Switzerland; 8 Paediatric Department, Cantonal Hospital of Winterthur, 8400 Winterthur, Switzerland; samuel.nef@ksw.ch; 9 Division of Child’s and Adolescent’s Surgery, Department of Pediatrics, Gynecology, and Obstetrics, University Center of Pediatric Surgery of Western Switzerland, 1004 Lausanne, Switzerland; oliver.sanchez@chuv.ch; 10 Nephrology Unit, University Children’s Hospital Zurich, 8008 Zurich, Switzerland; gi.sparta@bluewin.ch; 11 Division of Child’s and Adolescent’s Surgery, Department of Paediatrics, Gynecology, and Obstetrics, University Center of Paediatric Surgery of Western Switzerland, Geneva University Hospitals (HUG), 1205 Geneva, Switzerland; isabelle.andrieuvidal@hug.ch (I.V.); jacques.birraux@hug.ch (J.B.); 12 Paediatric Clinic, Widermeth Children’s Hospital, 2501 Biel/Bienne, Switzerland; rodo.vonvigier@szb-chb.ch

**Keywords:** urinary tract dilation, prenatal diagnosis, postnatal urinary tract dilatation, posterior urethral valves, ureteropelvic junction obstruction, vesicoureteric reflux, megaureter, ultrasound, VCUG

## Abstract

Urinary tract dilations (UTDs) are the most frequent prenatal renal anomaly. The spectrum of etiologies causing UTD ranges from mild spontaneously resolving obstruction to severe upper and lower urinary tract obstruction or reflux. The early recognition and management of these anomalies allows for improved renal endowment prenatally and ultimately better outcome for the child. The role of the general obstetrician and pediatrician is to recognize potential prenatal and postnatal cases addressed to their practice and to refer patients to specialized pediatric nephrology and urology centers with a sense of the urgency of such a referral. The aim of this paper is to offer clinical recommendations to clinicians regarding the management of neonates and children born with prenatally detected UTD, based on a consensus between Swiss pediatric nephrology centers. The aim is to give suggestions and recommendations based on the currently available literature regarding classifications and definitions of prenatal and postnatal UTD, etiologies, prenatal and postnatal renal function evaluation, investigations, antibiotic prophylaxis, and the need for referral to a pediatric nephrologist and/or urologist. The overarching goal of a systematic approach to UTD is to ultimately optimize kidney health during childhood and improve long-term renal function prognosis.

## 1. Introduction

**Figure 1:**
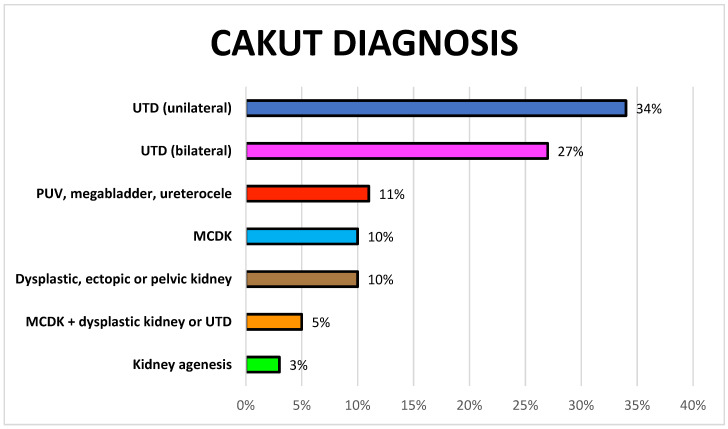
Types of congenital anomalies of the kidneys and urinary tract (CAKUTs) in a 10-year cohort studied in Geneva, Switzerland. Unilateral and bilateral pelvic dilation represent 61% of CAKUTs. UTD: urinary tract dilation; PUV: posterior urethral valves; MCKD: multicystic dysplastic kidney.

Congenital anomalies of the kidneys and urinary tract (CAKUTs) include a wide spectrum of malformations, representing 20–30% of all anomalies identified in the prenatal period [1,2] (Figure 1).

Nowadays, up to 60–85% of CAKUTs are prenatally diagnosed and occur at a frequency of 1:500 pregnancies [1], with approximately 80% detected by prenatal ultrasound [2,3]. Urinary tract dilations (UTDs) are the most frequent finding related to CAKUTs, which in turn, are the most common cause of renal failure and kidney transplantation in children [4].

The variability of these conditions is due to the complexity of kidney development, occurring throughout the full length of pregnancy: nephrogenesis begins at 6 weeks of gestation and concludes at 36 weeks of gestation [1]. Hence, altered nephrogenesis may lead to large variability in renal anomalies and, depending on their severity, result in low renal endowment (oligonephronia), similarly to what is observed in prematurity and neonates born small for gestational age. Nephron mass at birth predicts the risk of presenting chronic kidney disease (CKD), hypertension, and cardio-vascular disease in adulthood [5]. Thus, early recognition (nowadays mostly during the prenatal period) is key to improving management at birth. In 2014 [2], Nguyen et al. [2] published a novel multidisciplinary consensus classification with similar description criteria for UTD pre- and postnatally, with the aim of harmonizing findings [3,4,6].

This paper offers clinical recommendations for clinicians regarding the management of neonates and children born with prenatally detected UTD, based on a consensus between Swiss pediatric nephrology centers. While current practice is leaning towards incorporating more advanced diagnostic tools such as genetic testing in complex cases, these fall beyond the intended scope of this work, which aims to provide accessible, actionable guidance on clinical care for general pediatricians.

## 2. Methods and Consensus Preparation

The consensus recommendations were developed by members of the Swiss Working Group of Paediatric Nephrology (SAPN) and reviewed and approved by a pediatric radiology team, by the Swiss Society for Paediatric Urology (SwissPU), and by the Academy of Materno-Fetal Medicine of the Swiss Society of Obstetrics and Gynecology (SMFM), all experts in the management of pre- and postnatal UTD.

A panel, consisting of 10 pediatric nephrologists from SAPN, was created and met in May of 2022 to discuss the main points to be highlighted in the recommendations after a thorough literature review using PubMed, Cochrane, and Google Scholar. Grading of the evidence undertaken using the GRADE method (evidence quality: high, moderate, low, or very low; recommendation grading: weak or strong) [7] (Table A1). An outline of the manuscript with references to incorporate was created prior to the discussion. The main points that were discussed during the meeting were the following:

– Definitions of pre- and postnatal UTD and their most common etiologies.
– Recommended investigations to be performed postnatally and their timing based on the severity of prenatal UTD.
– When to use antibiotic prophylaxis.
– When to refer to a pediatric nephrologist and/or urologist.

A practice summary card, which was elaborated by the pediatric nephrology and pediatric radiology team of the Geneva University Hospitals in 2021, was modified accordingly and incorporated into the manuscript. The manuscript was sent to all representatives of the SAPN and to the presidents of SwissPU and SMFM for final review and approval.

## 3. Antenatal Urinary Tract Dilation (UTD)

Antenatal UTD account for 50 to 66% of anomalies detected by prenatal ultrasound, with a prevalence of 2.5 to 5% in the second trimester [5]. Although the presence of UTD may often be transient and physiological [8], it may also be associated with other CAKUTs and/or kidney damage [5,9].

### Prenatal Counselling

In case of persistent UTD (≥7 mm), prenatal counselling may be organized with a pediatric nephrologist. In severe situations where there is high suspicion of compromised renal function with increased risk of fetal morbi-mortality, a multidisciplinary approach is preferable with the presence of a urologist, an obstetrician, a neonatologist, and a geneticist.

Dedicated prenatal clinics are essential to explain and discuss the impact of the kidney anomaly with families, and to discuss management at birth and throughout childhood [10].

However, predictors of long-term renal prognosis are scarce; prenatal diagnosis has enabled the prompt management of kidney anomalies, improving early management [1]. The Swiss Maternity Health Care Program offers two ultrasound examinations to pregnant women: one in the first trimester and one in the second-trimester screening. The aim of the second-trimester ultrasound is to detect fetal anomalies, including kidney and urinary tract malformations. If any abnormality is found, a follow-up ultrasound on indication is necessary to evaluate amniotic fluid volume and fetal movements in the third trimester. Prenatal counseling can lead to further investigations in specific situations. An MRI can be useful to better evaluate anatomy, particularly in the context of a complex kidney or urinary tract anomaly, or in the presence of cysts. Genetic conditions resulting in CAKUTs are rare, but contribute to 16% of all kidney anomalies [11]. Most of these genes are transcription factors responsible for kidney development during pregnancy. Genetic analysis is offered mainly in case of a positive family history of severe kidney disease or in the presence of multiple fetal malformations [11].

## 4. Definitions and Classifications

### 4.1. Prenatal Pelvic Dilation

A normal anterior–posterior renal pelvis diameter (APRPD) is defined as a diameter of <4 mm between 16 and 27 weeks + 6 days of gestation and <7 mm at ≥28 weeks + 0 days of gestation.

A normal antenatal ultrasound (US) is defined by a normal APRPD (see previous statement) and the absence of central or peripheral calyceal dilation, normal parenchymal thickness and appearance, normal ureters, a normal bladder, and the absence of unexplained oligohydramnios.

Prenatally, a low-risk US is defined by a unilateral or bilateral APRPD of ≥7 mm and <10 mm at ≥28 weeks + 0 days and the absence of peripheral calyceal dilation, normal parenchymal thickness and appearance, the absence of ureteral dilation, and a normal appearance of the bladder. Single kidneys are excluded.

Prenatally, a high-risk US is defined by an APRPD of ≥7 mm before 28 weeks + 0 days and ≥10 mm at ≥28 weeks + 0 days and at least one of the following criteria: peripheral calyceal dilation, abnormal parenchymal thickness or appearance, ureteral dilation, abnormal appearance of the bladder, oligohydramnios.

Antenatal pelvic dilation is classified according to SFU criteria [12]. Six ultrasound findings are used to describe the urinary tract [2]:

- Anterior–posterior renal pelvic diameter (APRPD);
- Calyceal dilation with distinction between central and peripheral calyces;
- Renal parenchymal thickness;
- Renal parenchymal appearance;
- Bladder abnormalities;
- Ureteral abnormalities.

### 4.2. Etiologies of UTD

#### 4.2.1. UPJ (Ureteropelvic Junction) Obstruction

Ureteropelvic junction obstruction (UPJO) is defined as an obstruction of the flow of urine from the renal pelvis to the proximal ureter.

UPJO is most commonly a congenital condition caused by intrinsic stenosis due to abnormal muscle arrangement at the UPJ or a persistent fold in the ureter. A persistent blood vessel to the lower pole of the kidney causing compression of the UPJ can cause extrinsic obstruction (crossing vessel). In rare cases, UPJO may arise due to an obstructing stone or scarring following an infection, but this usually occurs later in childhood. The typical appearance on ultrasound is pelvic dilation without a dilated ureter. UPJO is found more commonly on the left side and is bilateral in up to 40% of cases [13]. In the presence of high-risk pelvic dilation with APRPD ≥ 15–20 mm, an MAG3-scan or functional uro-MRI should be performed after 1 month of age to help identify impaired function or delayed drainage, which, in some cases, may indicate obstruction.

In the majority of congenital cases, the condition is benign and does not require any intervention [14]. In more severe cases (for example, urodynamically significant outflow obstruction with asymmetry in renal function and/or recurrent urinary tract infection (UTI) or stones), temporary relief by internal ureteral derivation (double J) or external renal/ureteral derivation may be necessary to avoid renal pressure atrophy, with ultimate intervention consisting of pyeloplasty [15].

#### 4.2.2. Posterior Urethral Valves (PUVs)

Posterior urethral valves (PUVs) are defined as the presence of obstructing membranous folds within the lumen of the posterior urethra.

PUVs are the most common cause of lower urinary tract obstruction (LUTO) in male infants. Females may also present with LUTO (urethral atresia, persistent cloacae, prolapsing ureterocele), albeit less commonly than males.

The effect on renal function is variable and depends on the degree of renal dysplasia, which occurs secondary to the obstruction. However, PUVs are the most common CAKUTs causing CKD and ESKD in children [16]. Boys with suspicion of PUVs are mostly identified by antenatal US with bilateral pelvic dilation and hydroureter. Other signs can be bladder wall thickening, posterior urethral dilation, unilateral pelvic dilation, and oligohydramnios. After birth, the gold standard to identify PUVs is a voiding cystourethrogram (VCUG); however, only about half the patients with PUVs exhibit direct signs upon VCUG, and indirect signs (hypertrophied bladder neck, musculus interuretericus hypertrophy, and trabeculated appearance of the bladder wall) should prompt cystoscopy to confirm diagnosis [17,18]. PUVs can also manifest later in life with micturition symptoms; however, these presentations are out of the scope of this article.

The postnatal management of severe PUVs has to be immediate and may necessitate transfer to a tertiary care facility for the management of post-obstructive diuresis. The first step in treatment after stabilization of the child is to relieve the bladder outlet obstruction by inserting a urethral catheter, followed by cystoscopic valve ablation or vesicostomy.

#### 4.2.3. Vesicoureteric Reflux in Children (VUR)

Vesicoureteric reflux (VUR) is defined as the retrograde passage of urine from the bladder into one or both ureters, the renal pelvis, or both. VUR is graded according to the International Reflux Study Group (IRSG) classification.

VUR can be divided into two forms based on the underlying pathogenesis: primary VUR—the most common—or secondary VUR [19]. Primary VUR is typically a congenital condition caused by a dysfunctional ureterovesical junction (UVJ) due to a short intravesical ureter. The length of the intravesical ureter may be genetically determined and could explain the increased incidence in family members of patients with VUR.

Primary VUR may improve or even disappear during childhood because the ureters lengthen along with the growth of the child (particularly with the development of the pelvis, the change in the position of the bladder, and thus the geometry of the UVJ).

Secondary VUR is a result of abnormally high voiding pressure in the bladder due to obstructive uropathology such as PUVs, a neurogenic bladder, or bladder bowel dysfunction, particularly after toilet training [19].

The gold standard to detect and grade VUR is the VCUG [19]. Contrast-enhanced voiding urosonography (CEUS) may represent a future radiation-free alternative [20]; however, most contrast-enhanced products are still off-label for pediatric use in Switzerland [21]. Since the visualization of the urethra is more difficult in CEUS, it should not be used as a diagnostic tool in boys, but can be used to confirm suspected VUR in girls or in follow-up imaging. CEUS is a safe alternative with comparable sensitivity to detect VUR compared to VCUG, but requires an experienced specialized radiologist.

The International Reflux Study Group (IRSG) developed a classification system that grades VUR into five grades [6]:

- Grade I: Reflux limited to the non-dilated ureter.
- Grade II: Reflux into the pelvis and calyces without dilation.
- Grade III: Mild-to-moderate dilation of ureter, renal pelvis, and calyces.
- Grade IV: Moderate ureteral tortuosity and dilation of the pelvis and calyces.
- Grade V: Gross dilation of the ureter, pelvis, and calyces, loss of papillary impressions, ureteral tortuosity.

#### 4.2.4. Megaureter

Megaureter is defined by a dilation of the ureter of ≥7 mm visible on US. Megaureters are classified as obstructive, refluxing, obstructive/refluxing or non-refluxing/non-obstructive.

The dilation may involve the entire ureter or a part of it. Etiologies can be divided into primary (congenital) and secondary causes. Primary megaureter is a result of anatomic anomalies of the UVJ. Secondary megaureter is a result of anomalies of the bladder or urethra (neurogenic bladder, VUR, PUV).

Furthermore, the megaureter is classified as an obstructive, refluxing, obstructive, or refluxing or non-refluxing/non-obstructive megaureter. The diagnosis can be made by US. Further evaluation may consist of VCUG and MAG3 to distinguish the nature of the megaureter.

## 5. Evaluation of Perinatal Renal Function

Prenatal and perinatal renal function are difficult to predict, as no reliable renal marker of glomerular filtration rate (GFR) has been validated for these periods of significant physiological change. Table 1 describes perinatal markers of kidney function.

### 5.1. Evaluation of Fetal–Prenatal Renal Function

Our study group suggests performing at least two antenatal USs, the former in the second trimester to detect fetal anomalies, including kidney and urinary tract malformations, and the latter in the third trimester for the evaluation of amniotic fluid. *Evidence quality: moderate; recommendation: weak.*

Reliable in utero prognostic markers to predict renal function at birth are lacking. In severe bilateral CAKUTs, the estimation of fetal renal function and predicting postnatal function remains challenging. The principal tools available to the nephrologist are ultrasound examinations of the kidneys and urinary tract, amniotic fluid volume evaluation, and the biochemical evaluation of fetal urine.

Prenatal kidney ultrasound gives important information regarding parenchymal aspect, echogenicity, and kidney size, although it is not precise enough to predict renal function with accuracy.

After 14–16 weeks of gestation, amniotic fluid volume reflects fetal urine. Oligohydramnios is one of the most important prognostic parameters in evaluating kidney function in fetal life [23]. The biochemical evaluation of fetal urine (fetal urinary sodium, chloride, B2-microglobulin, cystatin C) has shown only partial reliability in predicting future renal outcome [24,25,26].

### 5.2. Evaluation of Postnatal Renal Function

Our study group recommends the measure of cystatin C in the cord blood of newborns with high-risk or bilateral urinary tract dilation, if available. If not, serial serum creatinine measurements are warranted. *Evidence quality: low; recommendation: strong.*

Most newborns with unilateral UTD will have a normal renal function at birth.

However, there are a few particularities about the neonatal kidney that the clinician must know to evaluate renal function properly [27,28]. Term and preterm infants have a low glomerular filtration rate that increases after birth, reaching normal values at the age of 12 months. Renal function at birth is inversely correlated with gestational age and weight [27,28]. Neonatal markers of renal function include: nadir serum creatinine, cystatin C, electrolyte handling, diuresis, and renal ultrasound.

The most frequently used biomarker of renal function, creatinine, can be difficult to interpret in the neonatal period as it crosses the placenta and reflects the mother’s value in the first 5 to 7 days of life [27,29,30].

Cystatin C is independent of sex, age, and muscle mass, and does not cross the placenta, or only to a very limited degree. It has been shown to be a specific and sensitive biomarker of GFR in neonates with UTD in particular [31]. Indeed, cystatin C measured in cord blood is predictive of the risk of CKD at one year of age [31]. Estimating GFR based on equations using endogenous markers (creatinine, cystatin C, urea) alone or combined has its limitations, particularly regarding the specificities of the neonatal population, and only a few methods have been developed and validated for this specific period of life [3,32,33].

Diuresis is also a marker of acute kidney injury and should be reported, taking into consideration that newborns present polyuria in the first days of life because of tubular immaturity: in this setting, a higher threshold of 1.5 to 2 mL/kg/h for normal urine output may be warranted [34,35].

Until now, there have been no reliable biomarkers that can be routinely utilized in risk stratification and decision-making for infants with pelvic dilation.

## 6. Postnatal Follow-Up

A normal anterior–posterior renal pelvis diameter (APRPD) is defined as a diameter of <10 mm postnatally (at >48 h of life).

Our study group recommends cord blood cystatin C measurement (or serial serum creatinine if cystatin C unavailable) for male newborns with a bilateral APRPD of ≥7 mm. *Evidence quality: low; recommendation: strong.*

Our study group recommends an urgent US at day 0 or 1 of life for male newborns with a bilateral APRPD of ≥7 mm. *Evidence quality: high; recommendation: strong.*

Of note, early US is known to underestimate dilation due to relative dehydration in newborns [36,37]. This first US is aimed at visualizing the kidney parenchyma and bladder aspect to help decide if urgent obstruction relief (through the insertion of a urethral catheter) is necessary.

Our study group recommends urgent placement of a urethral catheter at birth in cases of high suspicion of LUTO antenatally. *Evidence quality: high; recommendation: strong.*

Postnatally, if APRPD upon US is ≥15 mm alone or ≥10 mm with at least one of the stated criteria—peripheral calyceal dilation, abnormal parenchymal thickness or appearance, ureteral dilation, or abnormal appearance of the bladder—our study group recommends follow-up with US at 1 month of life with complimentary investigations to be discussed with a uro-nephrology team. *Evidence quality: moderate; recommendation: strong.*

Our study group suggests discussing the need for VCUG with a uro-nephrology team in cases of ureter dilation or bladder anomalies. *Evidence quality: moderate; recommendation: weak.*

Our study group recommends discussing all prenatal and postnatal cases presenting with a bladder anomaly, uni- or bilateral ureterocele, or solitary kidney with a uro-nephrology team. *Evidence quality: moderate; recommendation: strong.*

**Figure 2:**
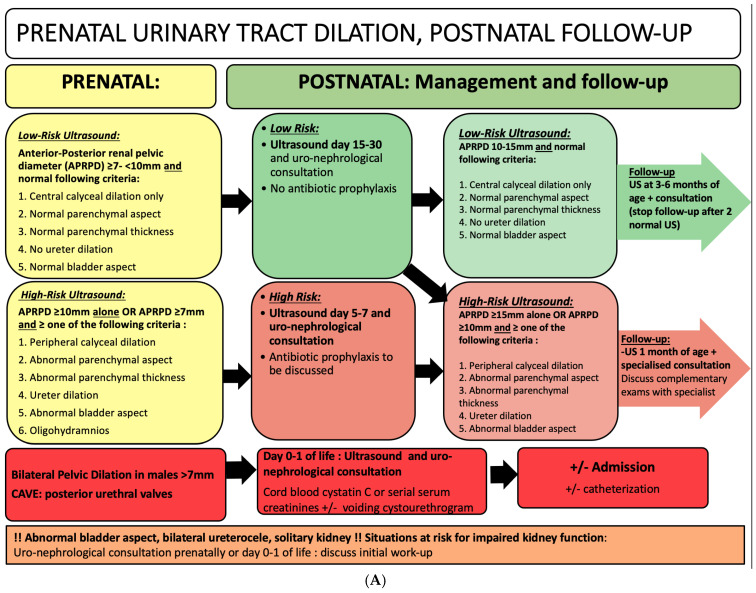

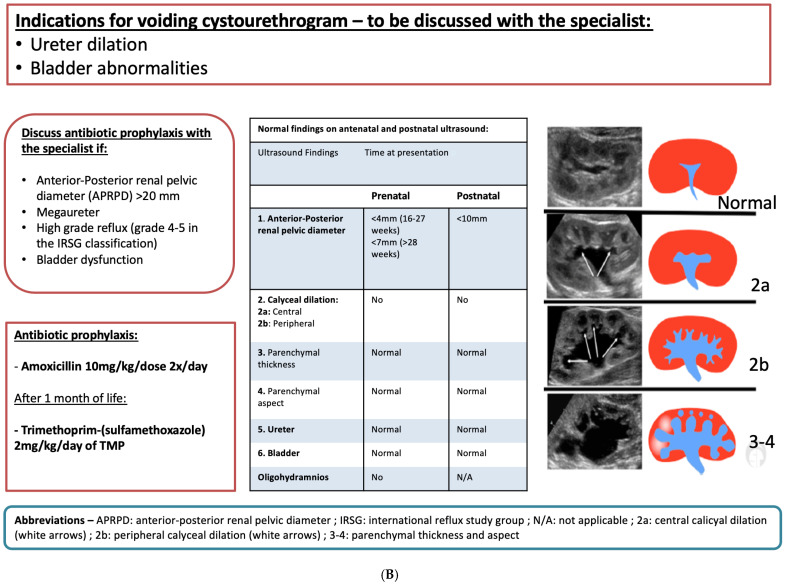
(**A**) Proposed postnatal follow-up for prenatal urinary tract dilation; (**B**) definitions.

Figure 2 summarizes the recommended postnatal follow-up of fetuses with prenatally diagnosed UTD.

### 6.1. VCUG

Although VCUG is performed less commonly than in the past, it remains the gold-standard exam for the detection of VUR [38]. Other indications for VCUG include the detection of ureterocele, bladder anomalies (e.g., diverticula), and urethral anomalies, such as PUVs. Infants with suspected bladder outlet obstruction should obtain an urgent VCUG.

VUR is the only uropathy in which the degree of the prenatal and postnatal urinary tract dilation does not correlate with increasing risk of pathology. In addition, the correlation between VUR grade and the severity of urinary tract dilation is poor [2]. Therefore, VUR cannot be excluded or diagnosed by simple ultrasonography.

To obtain valid results from VCUG, technical considerations are important:

– Lateral voiding views of the urethra after catheter removal and post-void views of the bladder are needed to formally recognize PUVs.
– Fill the bladder to its capacity. The volume of the urine removed post-catheter insertion should be reported and urine should be sent for analysis and culture, as there is a risk of UTI after VCUG.

### 6.2. Indication Tc-MAG3 Scan

The Tc-MAG3 Scan, also called dynamic renography, is one method of choice for functional renal assessment [39]. It is used to differentiate an obstruction requiring therapy from a dilation without relevant urinary obstruction and determine differential relative kidney function. The indication should be discussed on a case-by-case basis with the nephrologist/urologist.

Generally, the indications are as follows:

Suspicion of urodynamic relevant UPJO:
  ○ APRPD ≥ 15–20 mm.
  ○ Significant progression of pelvic dilation in the follow-up US.
  ○ Recurrent pain, UTI, or stones
Control after renal pyeloplasty.

MAG3 scintigraphy should not be performed earlier than 4–6 weeks of life because MAG3 is excreted primarily via proximal tubular secretion, which requires maturation of the system.

Magnetic Resonance Urography (MRU) is becoming increasingly important. It gives information not only regarding functional assessment, but also about the morphology of the urinary tract [2,40]. It may be used as an alternative to MAG3, but may require general anesthesia.

### 6.3. Indication DMSA Scintigraphy

DMSA scintigraphy is a diagnostic imaging method that uses dimercaptosuccinic acid (DMSA) to assess renal structure and function. It detects scars caused by infection or dysplastic kidneys [41]. Furthermore, it can be helpful in identifying ectopic or non-functional kidneys [42] and in detecting acute pyelonephritis [43] if other diagnostic methods are not suitable. Scintigraphy is not readily available in all centers.

## 7. Antibiotic Prophylaxis

Our study group does not recommend antibiotic prophylaxis in low-risk groups. *Evidence quality: high; recommendation: strong.*

Our study group suggests pondering the need for antibiotic prophylaxis in high-risk groups. This should be discussed with a nephrologist or urologist. *Evidence quality: moderate; recommendation: weak.*

Our study group suggests antibiotic prophylaxis in any of the following situations: APRPD > 20 mm prenatally or postnatally, megaureter ≥ 7 mm, high-grade (4 or 5) reflux, bladder anomaly. *Evidence quality: high; recommendation: weak.*

Our study group recommends the use of amoxicillin at 10 mg/kg/dose BID as antibiotic prophylaxis for newborns and trimethoprim (TM)–sulfamethoxazole at 2 mg/kg/dose of TM once daily after 1 month of life. *Evidence quality: moderate; recommendation: strong.*

Continuous antibiotic prophylaxis (CAP) for UTD remains subject to debate as it is mostly based on weak grading strength, as stated by several systematic reviews [44,45]. Although most authors agree that CAP for isolated low-risk UTD is not indicated [46,47], controversy remains regarding its benefits for patients with intermediate and high-grade UTD [46] with or without associated conditions. This results in a large heterogeneity in prescribing practices [48]. An ongoing randomized blinded placebo trial seems to show a significant benefit of treating high-risk UTD postnatally, at least in the first 6 months of life until initial work-up is completed [49]. For children with intermediate-risk UTD, the use of CAP should be at the discretion of the physician, based on individual risk evaluation and family preference.

Additionally, high-grade VUR (4 and 5) has been shown to be associated with risk of UTI recurrence [50] and renal scarring [51]. A significant risk reduction in UTI was seen with the use of CAP in a meta-analysis pooling all types of VUR [52], and in grade 3 to 5 VUR in a recent randomized controlled trial (PREDICT study group) [53]; however, this was at the price of an increased prevalence of multi-resistant bacteria and no reduction in new kidney scarring compared to the non-prophylaxis group [53], highlighting the importance of carefully assessing the decision to start CAP.

The Swiss recommendation for the type of CAP is amoxicillin (10 mg/kg 2 x/day) for the first month of life, followed by trimethoprim–sulfamethoxazole (TMP-SMX) (2 mg/kg/day or 1 mg/kg 2 x/day trimethoprim) if further indicated [54]. Nitrofurantoin is an alternative to TMP-SMX in cases of adverse effects or breakthrough infections (1 mg/kg 2 x/day). TMP-SMX and nitrofurantoin should be avoided in the neonatal period due to historical concerns of risk of kernicterus [55] and hemolytic anemia [56], respectively. Trimethoprim (2 mg/kg/day) alone, which can be used at birth, is not available in Switzerland and would require a request to health insurance for import from abroad.

## 8. When to Refer to a Nephrologist/Urologist

Our study group recommends performing a follow-up postnatal US between the 15th and 30th day of life in low-risk groups. *Evidence quality: moderate; recommendation: strong.*

Our study group suggests discontinuing follow-up in low-risk groups (excluding single kidney), after two normal USs at least 2 months apart, defined as an APRPD of <10 mm with a full bladder, the absence of peripheral calyceal dilation, normal parenchymal thickness and appearance, the absence of ureteral dilation, and normal appearance of the bladder. *Evidence quality: moderate; recommendation: weak.*

Postnatally, if APRPD is between 10 and 15 mm and there is an absence of peripheral calyceal dilation, normal parenchymal thickness and appearance, an absence of ureteral dilation, and a normal appearance of the bladder (excluding single kidney), our study group recommends follow-up with US between 3 and 6 months of life. *Evidence quality: high; recommendation: strong.*

Our study group recommends performing a postnatal US between the 5th and 7th day of life in high-risk groups. *Evidence quality: moderate; recommendation: strong.*

A child with a prenatal or postnatal high-risk UTD upon US should be followed by a pediatric nephrologist/urologist. All prenatally diagnosed kidney anomalies should be discussed with a pediatric nephrologist/urologist to anticipate immediate postnatal management. Parents should be offered multidisciplinary prenatal counselling with their obstetrician and a pediatric nephrologist and/or urologist. Other kidney anomalies, such as unilateral renal agenesis, pelvic kidney, or horseshoe kidney, should be addressed to a pediatric nephrologist postnatally for further follow-up.

In the absence of UTI and normal clinical course, follow-up may be discontinued in children presenting two normal postnatal USs at 3 to 6 months of age.

## 9. Long-Term Complications of UTD

Our study group recommends that children with persistent UTD should be followed periodically by a nephrologist and/or a urologist at least until the end of their growth, with blood pressure monitoring. *Evidence quality: high; recommendation: strong.*

The severity and the course of UTD evolution will define the long-term follow-up: in most of cases, prenatal UTD will resolve during childhood [9,57]. However, high-risk UTDs, such as those caused by severe VUR or PUVs, will need close follow-up by a pediatric nephrologist and/or urologist and may benefit from a transition program when the patient approaches adulthood.

Long-term follow-up may include the following:

- Uro-nephrology consultations until the end of the child’s growth;
- Yearly blood pressure control;
- Screening for albuminuria/proteinuria;
- Evaluation of GFR on a regular basis;
- Dietary consultation;
- Transition program upon adulthood.

## 10. Conclusions

This paper offers clinical recommendations by the Swiss Society of Pediatric Nephrologists, validated by the Swiss Society of Pediatric Urology and the Academy of Materno-Fetal Medicine of the Swiss Society of Obstetrics and Gynecology, on UTD, the most frequent prenatal kidney anomaly. The multidisciplinary collaboration between these societies aims to ensure the effective promotion of knowledge distribution and the adoption of these recommendations across all key specialties involved in the management of UTD. The practice summary card will serve as a tool to disseminate these recommendations through general pediatrics and various specialty conferences. Most cases of UTD spontaneously resolve by birth or in infancy without consequences on the child’s renal function. However, some UTDs may be related to an underlying, more severe anomaly. Decreased nephron mass resulting in abnormal kidney development during pregnancy will determine the progression of renal function over time. The standardization and expansion of obstetrical guidelines promotes the early recognition of cases requiring specialist referral for early management by obstetricians, ultimately leading to optimized follow-up during childhood, which can improve long-term renal function prognosis.

## Figures and Tables

**Table 1:** Evaluation of renal function at birth. GFR: Glomerular filtration rate, KDIGO: Kidney Disease Improving Global Outcomes.

Evaluation of Renal Function at Birth
Cystatin C at birth (cord blood), nadir and kinetic if available
Creatinine nadir and kinetic
Diuresis (neonatal acute kidney injury KDIGO criteria)
Estimating GFR through equations for newborns (not validated in this population): Mean GFR for a healthy term newborn between day of life 1 and 5 = 20 to 41 mL/min/1.73 m^2^ [22]
Blood gas (handling of bicarbonate) Blood biochemistry (handling of electrolytes) Urine biochemistry (concentration ability, albuminuria)

## List of Abbreviations

APRPD	Anterior–posterior renal pelvic diameter
CAKUTs	Congenital anomalies of the kidneys and urinary tracts
CAP	Continuous antibiotic prophylaxis
CEUS	Contrast-enhanced voiding urosonography
CKD	Chronic kidney disease
CysC	Cystatin C
DMSA	Dimercaptosuccinic acid
ESKD	End-stage kidney disease
GFR	Glomerular filtration rate
LUTO	Lower urinary tract obstruction
MAG3	99mTc-mercaptoacetyltriglycine
MCDK	Multicystic dysplastic kidney
PUV	Posterior urethral valves
TMP-SMX	Trimethoprim–sulfamethoxazole
UPJ	Ureteropelvic junction
UPJO	Ureteropelvic junction obstruction
US	Ultrasound
UTD	Urinary tract dilation
UTI	Urinary tract infection
UVJ	Ureterovesical junction
UVJO	Ureterovesical junction obstruction
VCUG	Voiding cystourethrogram
VUR	Vesico-ureteric reflux

## Appendix A

**Table A1:** GRADE system for evidence quality [7].

**Evidence quality**	
Very low	Case series or expert opinion
Low	Well-done observational studies
Moderate	Downgraded randomized controlled trials or upgraded observational studies
High	Randomized controlled trials or meta-analyses
**Grading system**	
Suggestion	Weak evidence
Recommendation	Moderate-to-strong evidence
